# Relative Comparison of Non-Invasive Brain Stimulation Methods for Modulating Deep Brain Targets*

**DOI:** 10.1109/EMBC48229.2022.9871476

**Published:** 2022-07-01

**Authors:** Emilė Radytė, Karen Wendt, Majid Memarian Sorkhabi, Jacinta O’Shea, Timothy Denison

**Affiliations:** Department of Psychiatry and the MRC Brain Network Dynamics Unit at the University of Oxford; MRC Brain Network Dynamics Unit, University of Oxford, Oxford, OX1 3TH, UK; MRC Brain Network Dynamics Unit, University of Oxford, Oxford, OX1 3TH, UK

## Abstract

This study models and investigates whether temporally interfering electric fields (TI EFs) could function as an effective non-invasive brain stimulation (NIBS) method for deep brain structure targeting in humans, relevant for psychiatric applications. Here, electric fields off- and on-target are modelled and compared with other common NIBS modalities (tACS, TMS). Additionally, local effects of the field strength are modelled on single-compartment neuronal models. While TI EFs are able to effectively reach deep brain targets, the ratio of off- to on-target stimulation remains high and comparable to other NIBS and may result in off-target neural blocks.

## I Introduction

Methodological advances in non-invasive brain stimulation (NIBS) aim to achieve less invasiveness, more focality and greater behavioural impact [1] relevant to treatment contexts. These goals must be balanced with ensuring safety and efficacy while using optimal energy settings and considering economic and practical feasibility [2].

Temporal interference (TI) stimulation is one of such promising methods, which is still novel, and claims to non-invasively target deep brain structures without affecting superficial structures, thus reducing potential off-target impacts. TI delivers stimulation from multiple current sources generating high frequency (> 1 kHz) stimuli, which only stimulate neurons where the two (or more) fields interfere [3]. Due to low-pass filtering properties of neural membranes, TI stimulation is not expected to cause neural activation outside of the interference target region, where only high frequency wave content is present [3] [4].

A recent modelling study [5], however, suggested that neuronal membranes most likely do respond to high-frequency stimulation in off-target regions during TI. Specifically, a “sandwich”-like pattern of neuronal firing can be expected as the distance from the target increases, progressing from phasic to tonic to alternating conduction blocks in the surrounding tissues. These conduction blocks off-target are not well understood but could disrupt normal firing patterns and potentially cause neuronal damage. This contrasts with previous research that indicated TI-induced activity at the target but none outside of it [3]. Understanding how TI affects neural tissue at- and off-target is a critical step to developing TI as a clinically applicable NIBS tool.

In addition to off-target effects, there are also some concerns about TI’s ability to reach the field strength necessary to modulate deep brain activity. Computational modelling has suggested that TI may be able to reach electric field strengths similar to those used in invasive deep brain stimulation (DBS), at >1.0 V/m, in rodents but not in humans (highest in the motor cortex at 0.57 V/m) [6]; leading to further studies that aim to instead modulate neural state changes at a much lower field strength of 0.2 V/m at the target [7].

With these challenges in mind, this study investigates current thresholds and optimal electrode montages needed to modulate a psychiatrically relevant deep brain target at the field strength comparable to DBS in humans. Here, the right lateral habenula was chosen as a novel target, since it’s been implicated in the pathophysiology and the potential treatment of major depressive disorder [8] [9], one of the most prevalent psychiatric illnesses with 258 million people suffering from it worldwide [10]. While the habenula is not a standard target in other NIBS or DBS methods, increasing research suggests it may be an extremely promising target for treatment in the future and is an excellent example of a psychiatrically relevant deep brain target that could be targeted with NIBS.

This study compares three different NIBS methods (TI, transcranial alternating current stimulation (tACS) and transcranial magnetic stimulation (TMS)) to better understand the behaviour of electric field distributions in the brain. Finally, a simple single-compartment computational model of a neuron is used to explore the modulatory and excitatory effects of the modelled electric fields on the neuronal membrane. Ultimately, this study aims to investigate the technical promise and challenges of TI for potentially applying it to psychiatric treatment targets in humans in the future.

## II Methods

### A Electrode montage and stimulation current optimization

Head models for TI, tACS and TMS simulations were created using the finite element method (FEM) with a realistic head model mesh derived from an example subject’s structural magnetic resonance scans in SimNIBS [11]. Default tissue conductivities were used (bone (0.010 S/m), scalp (0.465 S/m), gray matter (0.275 S/m), white matter (0.126 S/m), cerebrospinal fluid (1.654 S/m)). Simulation results were visualized with Gmsh and MATLAB, and all calculations were conducted in MATLAB.

Active electrode montages were determined by simulating electrodes on a 10-20 EEG cap that represents potential positions of electrodes on the scalp and compiles a matrix of the electric fields generated by every electrode, while keeping the return electrode constant, then optimized to reach a particular field strength at the target. Target (right lateral habenula) MNI coordinates were x = 4.8 (SD 0.39, range 4.3 to 6.0), y = − 24.1 (SD 0.55, range − 23.4 to − 25.3) and z = 2.2 (SD 0.47, range 1.4 to 3.5), all in millimetres [12].

For “low” intensity tACS and TI, an optimal montage was found to achieve close to 0.2 V/m at target (electric field strength for neuronal modulation). For “high” tACS and TI, as well as TMS, an optimal montage was then identified to achieve as close as possible to >1 V/m at target (electric field strength for neuronal stimulation). The maximum total current input for tACS and TI was limited to 10 mA for safety reasons based on previous research [13], and the montage was limited to a maximum of 2 active electrodes to validate and compare the experimental TI model with other NIBS modalities operating under shared limitations.

For tACS and TI modelling, standard square 5.0 cm x 5.0 cm gel electrodes were used with a 2.5 mm thickness. TMS was simulated using a standard, Magstim 70 mm figure-8 coil on SimNIBS, at a 4 mm distance to the scalp. The specific positions in the 10-20 electrode system for each of the montages are shown in Table 1.

### B Temporally Interfering Electric Field Simulation

SimNIBS can only model maximal EF strengths at one particular time point rather than their variation with time, therefore maximal EF strengths of the two electrode pairs involved in TI were modelled separately. Then, at each MNI coordinate of interest, the maximum envelope amplitude resulting from TI was calculated to evaluate target, maximum off-target, and average off-target effects. These results therefore analyse the effects of maximum field strength in particular positions of the brain allowing for a good understanding of impacts that the strongest effects, including side effects, observed at a particular position would be. The electric field resulting from the interference of the two waves was calculated by deriving the maximal resulting envelope amplitude using the amplitude modulation (AM) concept and the procedure first presented in [3]:

(1) $$\begin{array}{l} \;\left|{\vec{E_{AM}}}^{\text{Max}}(\vec{r})\right|=\\ \{\begin{array}{l} 2\left|\vec{E_{2}}(\vec{r})\right|,\;If\;\left|\vec{E_{2}}(\vec{r})\right|<\left|\vec{E_{1}}(\vec{r})\right|\text{cos}\;(\alpha )\\ \frac{2\left|\vec{E_{2}}(\vec{r})\times \left(\vec{E_{1}}(\vec{r})-\vec{E_{2}}(\vec{r})\right)\right|}{\left|\vec{E_{1}}(\vec{r})-\vec{E_{2}}(\vec{r})\right|},\;\text{otherwise} \end{array} \end{array}$$

which builds on the computations of each of the electric fields,

(2) $$\left|{\vec{E}}_{AM}(\vec{n},\vec{r})\right|=\left|\left|\left(\vec{E_{1}}(\vec{r})+\vec{E_{2}}(\vec{r})\right)\cdot \vec{n}\right|-\left(\left|\vec{E_{1}}(\vec{r})-\vec{E_{2}}(\vec{r})\cdot \vec{n}\right|\right)\right|$$

where $\vec{E_{1}}$ and $\vec{E_{2}}$ are electric fields generated by the first and second electrode pair in location $\vec{r}$ (x,y,z), *α* is the angle between $\vec{E_{1}}\;$ and $\vec{E_{2}}$, and $\vec{n}$ is the unit vector of the normal to the surface. This results in the maximal envelope amplitude along the normal at the location (x,y,z) and can be used to investigate smaller scale changes impacting neurons at (x,y,z).

### C Single-compartment neuron models

The Hodgkin and Huxley (HH) single-compartment model, based on the landmark squid giant axon characteristics considers leakage current, a transient Na+ current, and a delayed-rectified K+ current. This study used an adapted HH type neuron model that includes properties of other ion channels. This captures the properties of thalamic neurons in the rat somatosensory cortex [14], and is more representative of mammalian neurons at a single-compartment level. This type of model is most helpful in understanding the dynamics of how an action potential is generated over time in a highly simplified, excitatory regular spiking (RS) [15] neuron model.

A 2 kHz and a 2.01 kHz sine wave were applied to the adapted HH model separately, which would be similar to the type of stimulation experienced by superficial, or off-target, neurons. Then, both sine waves were applied simultaneously, to show how their interference would impact the generation of an action potential (neural activation) in this simple model where the stimuli interfere. Since this model does not represent habenular neuron morphology, it is merely used to illustrate possible dynamics, not estimate exact effects. Finally, an additional single pulse square wave was applied to test the hypothesis of an off-target conduction block.

## III Results and discussion

### A Modelled TI EF field strengths

For tACS and TI simulations, two optimisations were designed as described in the Methods. They are abbreviated to low TI/tACS and high TI/tACS accordingly. The resulting electrode positions are displayed in Table 1.

**Fig. 1:**
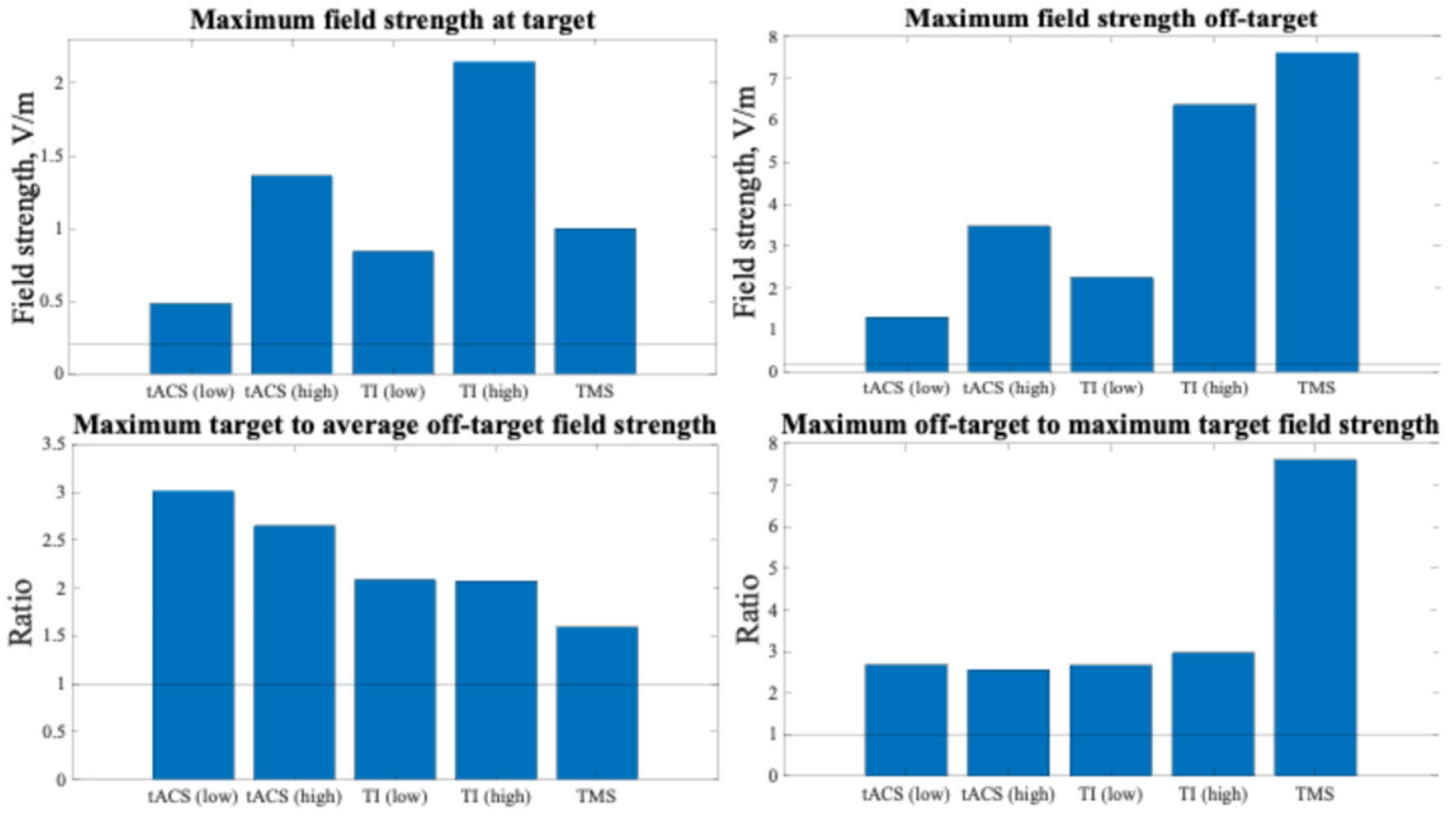
Across studied NIBS modalities, high TI is able to reach the highest field strength at the lateral habenula target but also has the highest field strength off-target; its ratio of off-target to target field strengths is comparable to other NIBS modalities. a) Shows the maximum field strength reached at the target for each of the NIBS modalities. b) Shows the maximum electric field strength off-target across NIBS modalities. c) Shows the maximum field strength at the target to average off-target ratios across NIBS modalities. d) Shows the maximum field strength off-target to target ratios across NIBS modalities. For a) and b): The horizontal line at 0.2 V/m marks the threshold for expected neural membrane modulation effects. For c) and d): 1 marks the equivalence threshold; all modalities performing above 1 have proportionally higher off-target than on-target field strengths.

From these electrode positions, average off-target and maximum on- and off-target field strengths were calculated. Comparing those strengths shows that across studied NIBS modalities, high TI is able to reach the highest field strength at the deep lateral habenula target but also has very high field strengths off-target; its ratio of off-target to target field strengths is comparable to other NIBS modalities. Note that off-target field strengths for TI are assumed to not have an effect on neurons because of their low-pass filtering properties that neglect high frequency wave content. Field strengths across these scenarios are summarised in Fig. 1 below.

### B HH neuron response to TI EF strengths at- and off-target

**Fig 2:**
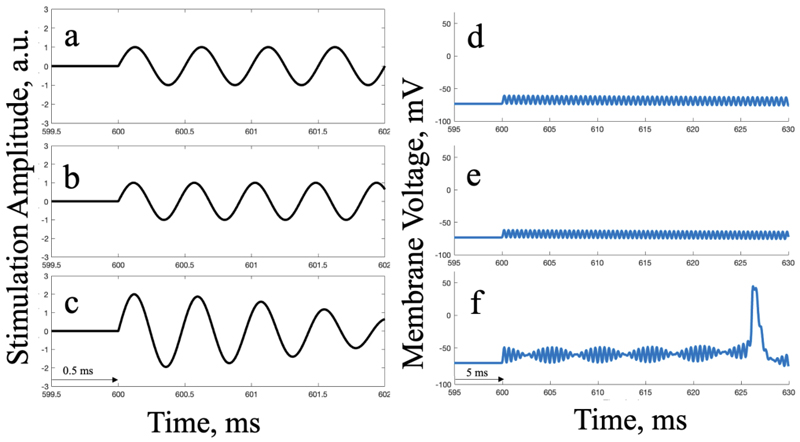
Under TI hypotheses, TI EFs do not initiate action potentials in off-target regions but generate an action potential in the interference region at the target in the single-compartment RS HH model. The panels on the left (a-c) show a short time window of the stimulation pulse (in a.u.) for a 2 kHz, a 2.01 kHz, and interfering 2.01 and 2 kHz sine waves, respectively. The panels on the right show the hypothetical membrane voltage response to the respective stimulus (in mV), over time. Panels d) and e) demonstrate the no-response hypothesis to the respective wave content (a and b) off-target; f) demonstrates neural activation in response to wave interference (c) at the target.

Field strengths do not capture the full effects of stimulation on cells across the brain. In order to better understand the effect expected at the single neuron level resulting from TI stimulation, a simple single-compartment HH type neuron model was used as described in the Methods. Fig. 2 below demonstrates the TI hypothesis on this model.

**Fig. 3:**
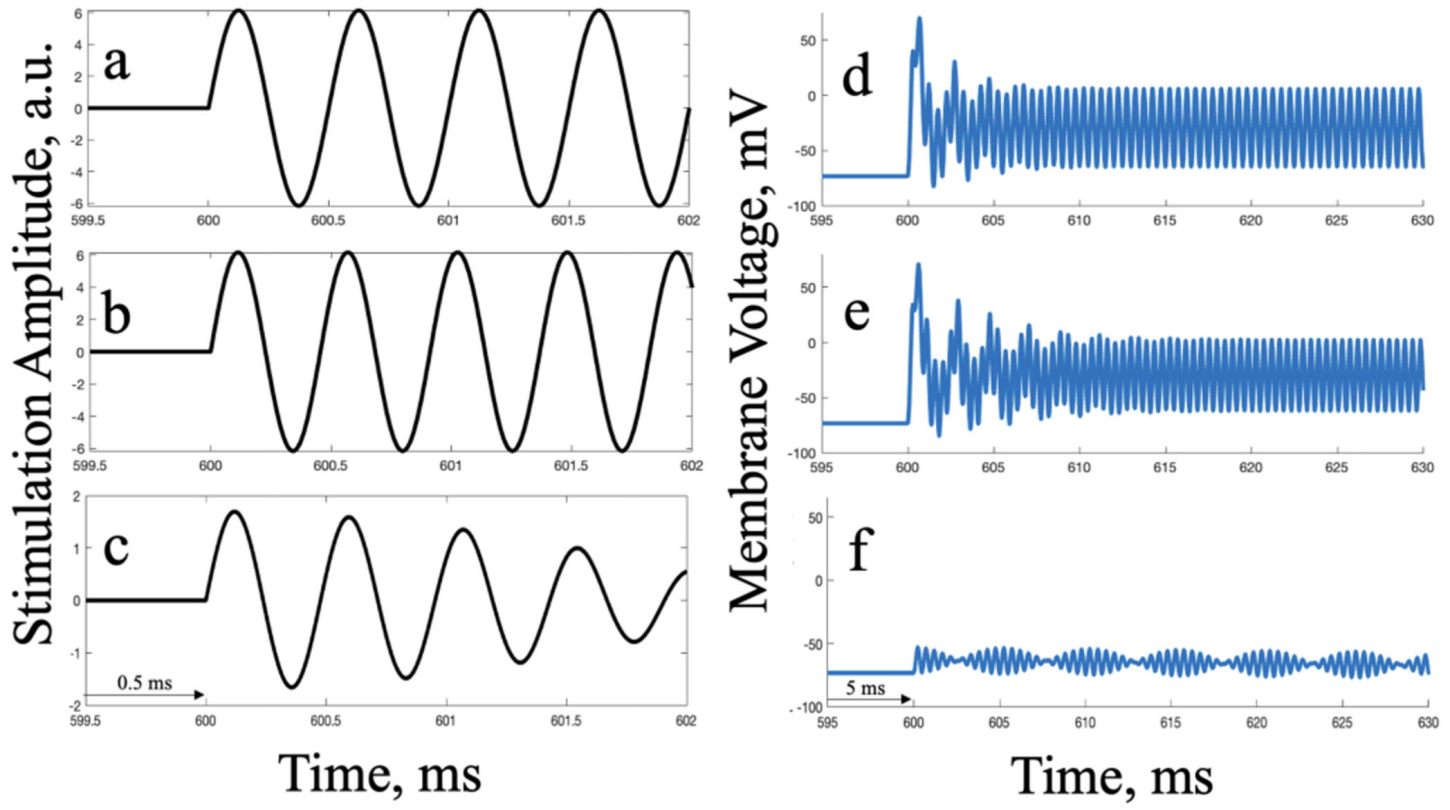
At maximum field strengths, high TI generates action potentials in off-target (non-interfering) regions but not in the lateral habenular (interfering) region in the single-compartment RS HH model. The panels on the left (a-c) show a short time window of the stimulation pulse (in a.u.) for a 2 kHz, a 2.01 kHz, and interfering 2.01 and 2 kHz sine waves, respectively. The panels on the right show the modelled membrane voltage response to the respective stimulus (in mV), over time. Panels d) and e) show the initiation of an action potential and subsequent oscillation in response to the respective high frequency wave content (a and b) off-target; f) demonstrates neural modulation in response to wave interference (c) in the targeted region.

The hypotheses are tested with acquired electric field strengths from realistic simulations. This, while a simplified model, is merely aimed at giving an intuition on how electric field strengths at target neurons could compare to those off-target, but are in no way used to argue their accuracy in human models, as these cellular models are too simplistic. Figure 3 shows that while high TI is able to modulate deep brain targets at maximal strengths, it also generates off-target action potentials.

Effect summary of experimental EF strengths on RS HH neuron single-compartment models is shown in Table 2.

### C Other effects on single-compartment models

**Fig. 4:**
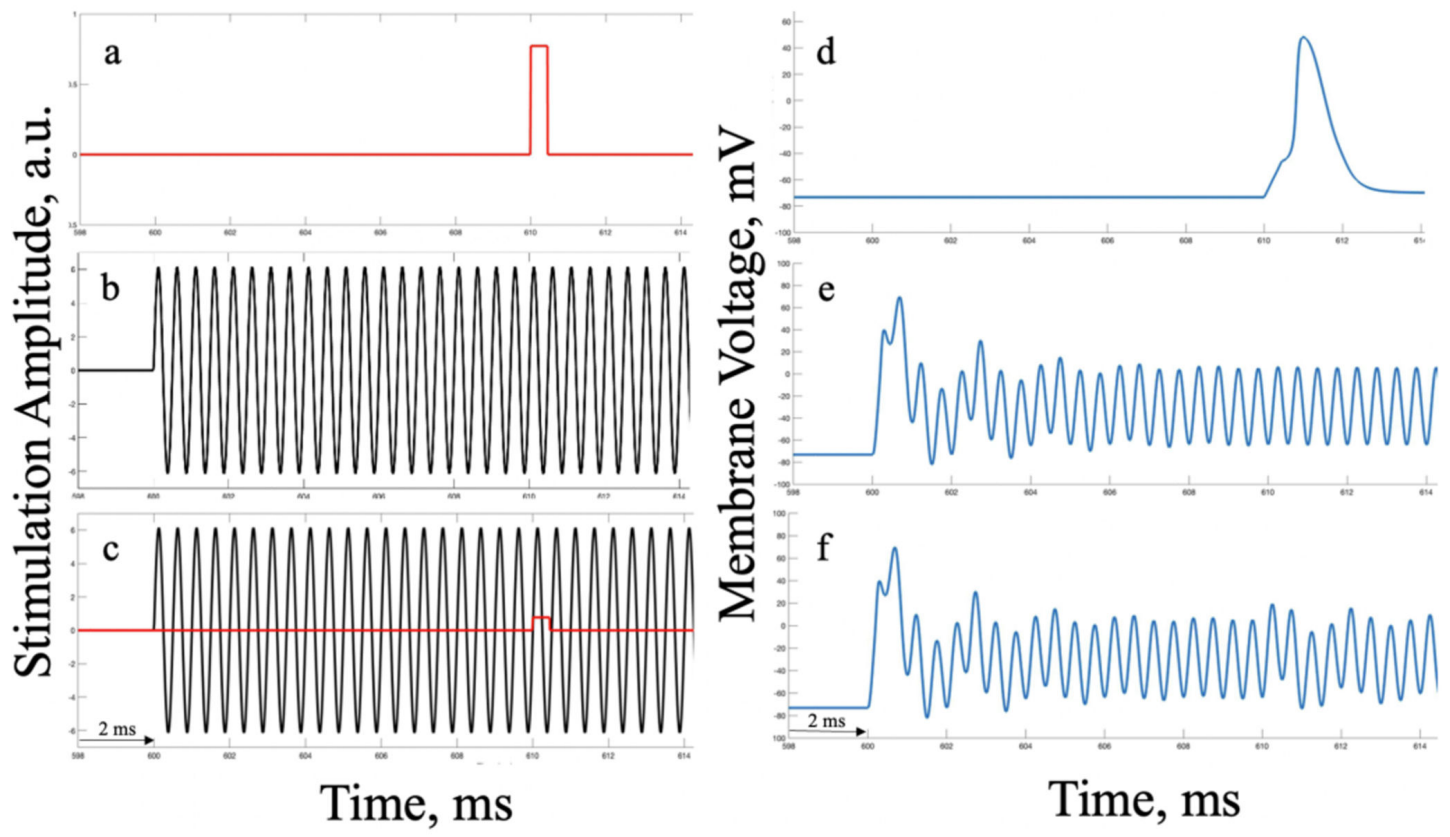
External stimulation effect in addition to 2 kHz sine waves under high TI field strengths on the single-compartment RF HH model. Panels on the left (a-c) show a short time window of the applied stimulation pulses (in a.u.), panels on the right show the membrane voltage response to the corresponding stimuli (in mV), over time. Panels a) and d): simple square wave stimulation on a resting neuron; b) and e): stimulation from a 2 kHz sine wave (off-target) under high TI field strengths; c) and f): stimulation from a square wave pulse applied (from Fig 4.a) 10 ms after the application of a 2 kHz sine wave pulse (off-target TI) (from Fig 4.b). Induced electric field under TI stimulation appears to generate a conduction block in off-target regions.

The effect of neural modulation and stimulation is not limited to action potential initiation and propagation. It has been recently proposed that off-target effects of TI EFs could include a conduction block [5]. A conduction block describes a condition where another action potential cannot be initiated in response to additional stimulation. This was explored by applying a single, brief square current pulse that is able to initiate an action potential in a resting neuron (Fig 4.a, d) to an off-target cell under 2 kHz TI stimulation (Fig 4.b, e). As shown in Fig. 4.c, f, under the influence of a high-frequency wave in off-target, superficial regions of the brain, cells do not appear to generate action potentials in response to other stimulatory inputs, suggesting that a conduction block leading to neural blocking is in place, even if there is some variation in the resulting waveform, as shown below.

As the applied TI EF increases, the threshold for an external stimulus to initiate an action potential scales accordingly. Importantly, this suggests that TI can have off-target effects beyond stimulation and/or modulation that could lead to unmeasured side effects of clinical significance down the line.

## IV Limitations

The models used in this study are a simplification of the effects we would expect in the context of realistic brain models and psychiatric treatment. Further simulations with multi-compartmental models could add more complexity and more biophysically realistic results for future applications that would consider the orientation of electric field vectors along neuronal morphologies, as well as the activating function.

Critically, neuronal activity can generate response patterns more complex than modulation and activation when affected by external inputs. One of such effects is a conduction block, expected in off-target areas in TI stimulation, which cannot be properly accounted for without a better understanding of the focality of TI stimulation. Yet, some preliminary results shown here demonstrate that, in simple models, a conduction block off-target may be present. This work does not account for the fact that changing external stimulus waveform parameters, such as pulse amplitude and width, could result in different response patterns than those observed.

While this study was restricted to montages with two electrode pairs and does not include models for other NIBS methods, such as focused ultrasound, more complex montages with steering properties have been proposed [16]. This should be investigated in future studies.

## V Conclusion

This study showed what induced field strengths could be expected in the brain for on-target and off-target TI EF applications, when compared to other conventional NIBS methods that aim to reach a psychiatrically relevant deep brain target, the lateral habenula. It showed that the possibility of neural conduction blocks off-target is expected in at least some off-target areas under TI EF stimulation. Further research should interrogate the distribution, spread, and importance of these neural blocks as well as their effect dependence on neuronal morphologies at the local neuronal level before these methods can begin to be considered for safety and efficacy when applied in clinical contexts.

## Figures and Tables

**Table I:** Optimal electrode positions in the 10-20 EEG system and input current strengths (in mA) for specific modelling aims.

Optimisation aim	Optimal 10-20 electrode position (input current strength)
tACS optimised for 0.2 V/m field strength at target	FT10 (-2.598 mA) / Iz (2.598 mA)
TI EF envelopes optimised for 0.2 V/m at target	F9 (-2.913 mA) / P9 (2.913 mA); F10 (-2.913 mA) / POz (2.913 mA)
tACS optimised for > 1V/m field strength at target	F6 (-5.000 mA) / PO3 (5.000 mA)
TI EF envelopes optimised for > 1V/m at target	F6 (-5.000 mA) / O1 (5.000 mA); F4 (-5.000 mA) / PO3 (5.000 mA)
TMS optimized for > 1V/m at target	C6 (2.00e6 A/s (di/dt))

**Table II:** Effect summary on RS HH single-compartment neuron models of electric field strength from full head model simulations

Modality	Neuron membrane voltage variation at maximum field strength at the target	Neuron membrane voltage variation at maximum field strength off-target	Neuron membrane voltage variation at average field strength off-target
TI EF envelopes optimised for 0.2 V/m at target	Interfering, low amplitude oscillatory activity	Low amplitude oscillatory activity	Low amplitude oscillatory activity
TI EF envelopes optimised for >1 V/m at target	Interfering, higher amplitude oscillatory activity	Action potential, then high amplitude oscillatory activity, conduction block	Low amplitude oscillatory activity

## Clinical Relevance

This study builds on earlier work and demonstrates some of the challenges –such as off-target conduction blocks– of applying TI EFs for targeting deep brain structures important in understanding the potential of treating neuropsychiatric conditions in the future.
